# Should less motion sensitive T2-weighted BLADE TSE replace Cartesian TSE for female pelvic MRI?

**DOI:** 10.1007/s13244-012-0193-9

**Published:** 2012-09-26

**Authors:** Johannes M. Froehlich, Thierry Metens, Bianka Chilla, Nik Hauser, Markus Klarhoefer, Rahel A. Kubik-Huch

**Affiliations:** 1 Department of Radiology, Kantonsspital Baden, Baden, 5404 Switzerland; 2 MRI Unit, Hôpital Erasme, Université Libre de Bruxelles, Brussels, Belgium; 3 Department of Gynecology, Kantonsspital Baden, Baden, 5404 Switzerland; 4 Siemens Healthcare, Freilagerstr. 40, Zürich, 8047 Switzerland

**Keywords:** Pelvis, Female, Magnetic resonance imaging, Image processing, Artefacts

## Abstract

**Objectives:**

To prospectively compare the diagnostic performance of a non-Cartesian k-space sampling T2-weighted TSE BLADE sequence with a conventional T2-weighted TSE sequence in female pelvic organs.

**Methods:**

Forty-seven patients with sonographically indeterminate adnexal masses or uterine lesions underwent sagittal BLADE and conventional TSE at 1.5 T after glucagon administration. Two radiologists independently determined their preferred sequence by rating: overall image diagnostic quality, conspicuity of the zonal anatomy and delineation of pathologies of the uterus and cervix, presence of artefacts, and of fluid in the pouch of Douglas (Wilcoxon signed rank test). Signal-to noise ratios (SNRs) and contrast-to-noise ratios (CNRs) were measured for the myometrium versus the rectus abdominis muscle (Student’s *t*-test).

**Results:**

BLADE significantly (*p* < 0.0001) reduced motion and ghosting artefacts and showed improved conspicuity (*p* = 0.3/0.24), but overall image quality did not differ significantly (inter-observer agreement BLADE κ = 0.89; TSE κ = 0.84). In the majority of cases (53.2 % vs 59.6 %, respectively, κ = 0.82) radiologists preferred conventional TSE due to better image contrast (*p* < 0.0001) and visibility of free pelvic fluid (*p* ≤ 0.0001). SNR (TSE 57.5 ± 37.7; BLADE 16.6 ± 12.2) and CNR (TSE 40.4 ± 33.5; BLADE 7.2 ± 8.8) were significantly higher on conventional TSE (*p* < 0.0001).

**Conclusions:**

Although BLADE reduces motion artefacts and provides a clearer delineation of uterine zonal anatomy compared with conventional TSE, this comes at the expense of overall contrast.

***Main Messages*:**

• *Use of BLADE may reduce T2 contrast and thus visibility of free pelvic fluid or cystic structures*

• *Non-Cartesian sampling of k-space such as BLADE is beneficial due to less motion sensitivity*

• *BLADE provides clearer delineation and conspicuity of uterine zonal anatomy on pelvic MRIs*

## Introduction

T2-weighted contrast plays an important role in magnetic resonance imaging (MRI) of the female pelvis. T2-weighted imaging has been proposed as part of a basic MRI protocol for work-up of pathologies of the female genital organs, i.e. sonographically indeterminate adnexal masses [1] or staging of endometrial cancer with MRI [2]. In pelvic MRI, respiration, cardiac motion and intestinal peristalsis often produce movement or ghosting artefacts which may result in poorly defined organ contours and reduced detection of pathological lesions. T2-weighted sequences are particularly prone to motion artefacts due to long imaging times. Various techniques have been proposed to overcome these limitations, such as the use of anti-peristaltic drugs, fasting, meticulous patient fixation and the use of single-shot pulse sequences; however, they all suffer limitations in clinical routine.

**Fig. 1:**
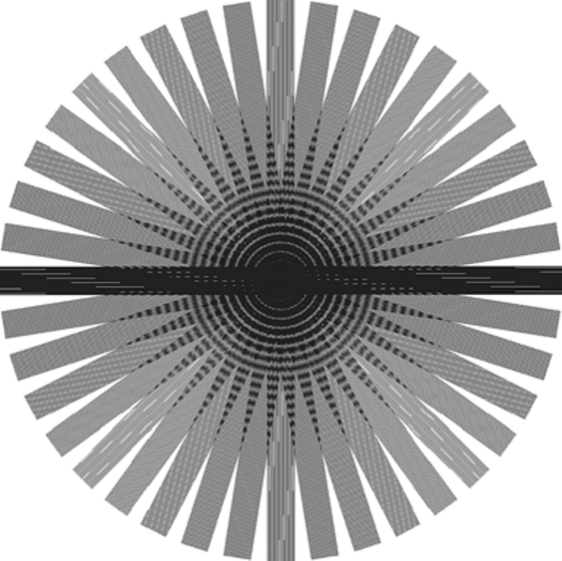
Illustration of BLADE k-space data acquisition. Each blade contains 19 phase-encoding lines (turbo factor 19). This drawing shows a complete set of trajectories composed of 20 rotated strips for BLADE data

The multiple over-sampling of central k-space using periodically rotated overlapping parallel lines with enhanced reconstruction (PROPELLER) offers significant advantages over other methods for patient motion correction, as it corrects for two types of motion: in-plane rotation and translation. BLADE is Siemens’ proprietary name for this technical approach [3, 4]. Unlike rectilinear k-space sampling, BLADE uses a turbo spin echo (TSE) sequence to acquire multiple echo trains (BLADEs) in a rotating, partially overlapping fashion (Fig. 1). This technique is less susceptible not only to bulk motion but also to flow-related artefacts from vessels, and has been evaluated for replacing sequences with conventional Cartesian k-space acquisition in the brain [5]. Recent work by Lane et al. [6], Koyama et al. [7], Fujimoto et al. [8] and Haneder et al. [9] demonstrated the potential of this technique to improve image quality in the small pelvis by reducing motion artefacts resulting from bowel peristalsis, breathing and abdominal wall motion. However, these authors focused primarily on visual and qualitative assessment of images. The aim of the present study was to prospectively compare overall image quality, contrast, and diagnostic information of the recently implemented T2-weighted BLADE TSE sequence with the conventional Cartesian T2-weighted TSE sequence in female pelvic organs.

## Materials and methods

### Patients

Forty-seven consecutive patients (mean age 47.2 years; range 19–88 years; 28 pre-menopausal, 2 peri-menopausal, 17 post-menopausal) with indeterminate adnexal masses or uterine lesions at ultrasonography were included in this prospective clinical trial. The study was approved by the institutional review board and all patients gave written informed consent. A subgroup of patients with suspected ovarian pathology was included in a previous study addressing the impact of MRI on diagnosis and therapy of sonographically indeterminate adnexal masses [10].

### MRI protocol

MRI was performed in supine position on a 1.5-T whole-body scanner (Magnetom Avanto; Siemens Healthcare, Erlangen, Germany) using a six-channel phased array body coil. Our routine clinical abdominal and pelvic protocol included several three-plane pulse sequences with differing image weightings including steady-state free precession (TrueFisp), T1-weighted two-dimensional (2D) gradient echo, diffusion, and several T1- and T2-weighted TSE sequences. Our clinical protocol also includes a conventional Cartesian sagittal T2-weighted TSE sequence (TR 4,650 ms, TE 101 ms, refocusing flip-angle 175°, turbo factor 13; echo spacing 14.4 ms, field of view [FOV] 25 cm, matrix size 256 × 512 voxels, slice thickness 4 mm (gap 30 %), GRAPPA (generalised autocalibrating partially parallel acquisition) factor 2, bandwidth 130 Hz/pixel; acquisition time 3:49 min). Sagittal T2-weighted BLADE TSE images were acquired for study purposes (TR 4,500 ms, TE 86 ms, refocusing flip-angle 145°, turbo factor 19, echo spacing 8.56 ms, FOV 25 cm, matrix size 384 × 384 voxels, slice thickness 4 mm (gap 30 %), GRAPPA factor 2, bandwidth 260 Hz/pixel, total scan time 3:11 min). The BLADE-specific imaging parameters were: 100 % blade coverage and 20 blades. The rotation angles and number of blades were chosen to fully cover k-space [5].

The acquisition order of the BLADE and conventional TSE sequences was randomised so that the patient was not aware of the order of sequences and, in order to exclude influences from spasmolytic drug. Glucagon (1.0mg) was administered intravenously as a spasmolytic agent (Glucagen®; Novo Nordisk, Switzerland) shortly before the first of the T2-weighted sagittal acquisitions [11]. Spasmolysis thus occurred under randomised conditions without influencing overall image quality. Both sequences were acquired without respiratory triggering and with an identical number of slices covering the entire pelvis, and parameters were chosen to keep acquisition lengths and echo times as similar as possible.

### Qualitative image analysis

All image data sets were transferred to a Picture Archiving and Communication System (PACS) workstation for image analysis (Centricity® PACS; GE Healthcare, Milwaukee, WI, USA). MR images were evaluated separately by two radiologists with 17 and 8 years of experience in female imaging respectively. Radiologists were blinded to the acquisition modes and temporal course of the MR protocol, and to other patient data such as transvaginal ultrasound (TVUS) and medical history.

All images were presented in random order to each of the readers and were evaluated on a five-point scale for various criteria defining image quality. Overall image diagnostic quality of normal anatomy and potential pathologies (if present) within the uterus, cervix, ovaries, intestine or bladder were ranked according to the following scale: 1 = non-diagnostic, 2 = poor, 3 = fair yet still diagnostic, 4 = good, 5 = excellent. The severity of ghosting or streak artefacts was scored as follows: 1 = profound, non-diagnostic, 2 = severe yet still diagnostic, 3 = moderate, disturbing but diagnostic 4 = visible, non-disturbing, 5 = imperceptible. Zonal anatomy of the uterus including cervix delineation and conspicuity was assessed on a five-point scale according to: 1 = major blurring, 2 = inhomogeneous blurring, 3 = moderate blurring but diagnostic, 4 = slightly visible blurring, 5 = sharp margins of structures. Readers were asked to rate presence of fluid in the pouch of Douglas and its visibility. Finally, readers were asked to indicate their preferred sequence (sagittal T2-weighted BLADE TSE or sagittal T2-weighted TSE) taking into account the diagnostic performance and overall contrast. If no difference was visible equal ranking was allowed.

### Quantitative analysis of contrast

The signal-to-noise ratios (SNRs) and contrast-to-noise ratios (CNRs) of the myometrium and rectus abdominis muscle were determined on a dedicated workstation (Leonardo; Siemens Healthcare Systems, Erlangen, Germany). Regions of interest (ROIs) of the same size were placed on images of both sequences by the readers. ROIs were drawn as large as possible, excluding areas with lesions, pronounced artefacts or evident tissue inhomogeneities. The noise ROIs were drawn in artefact-free air space on the same slice and height as the myometrium. CNRs were calculated as the signal intensity of the myometrium minus the signal of the muscle tissue divided by the standard deviation of the noise.

### Statistical analysis

A Wilcoxon signed-ranked test (two-tails of probabilities; normal distribution) was used to assess qualitative scores differences between the two sequences. A two-sided paired Student *t*-test was used to assess pulse sequence dependent differences for both SNRs and CNRs after testing for normal distribution; a *p* value of less than 0.05 was considered indicative of a significant difference. Statistical analyses were performed using SPSS 15.0 for Windows (SPSS, Chicago, IL, USA). Inter-observer agreement for the qualitative rating was calculated using κ-statistics. Kappa scores (κ) of 0.41-0.60, 0.61-0.80, and ≥0.80 were regarded to be indicative of moderate, good, and excellent agreement, respectively.

## Results

Malignant pathologies were identified in eight cases (endometrial cancer *n* = 3, cervical adenocarcinoma *n* = 1, ovarian cancer *n* = 4). Benign gynaecological pathologies were identified in 36 of the remaining 39 patients (leiomyoma *n* = 8, teratoma *n* = 6, endometrioma *n* = 9, cystadenoma or/and follicular cysts *n* = 9, sactosalpinx *n* = 1, fibrothecoma *n* = 3). No evident pathology was detectable in three patients. The majority of cases underwent surgery (43 patients) and had histopathological confirmation of diagnosis.

Both sequences of interest were acquired according to the study protocol in all patients. One drawback of BLADE sequence was the prolonged time (approximately 3 min) needed for post-processing before images were displayed, which delayed planning for the subsequent axial-oblique planes of the long axis of the uterus. In general, the duration of post-processing is dependent on patient size, motion, BLADE parameters, GRAPPA factors and computing power.

### Qualitative comparison between T2-weighted conventional TSE and BLADE TSE images

Overall image quality including diagnostic performance and visibility of pathological lesions was rated equal on conventional and BLADE TSE. Inter-observer agreement for overall image quality reached κ = 0.89 for BLADE TSE and κ = 0.84 for conventional TSE, indicating excellent agreement. The qualitative assessment showed a significantly reduced number of ghosting artefacts (*p* < 0.0001) and slightly improved conspicuity of the uterine contours for BLADE (*p* = 0.303/0.238). Overall contrast and the visibility of fluid were rated significantly better on conventional TSE (*p* ≤ 0.0001). Both observers preferred the conventional T2-weighted TSE pulse sequence in the majority of cases (59.6 % and 53.2 %, respectively; κ = 0.82; agreement of both observers in 89.4 %), primarily due to the better visibility of fluid (21/22 cases, respectively). Nevertheless, T2-weighted TSE BLADE was preferred in 19.1 % and 14.9 % of cases, respectively. Detailed results with statistics are summarised in Table 1.

**Table 1:** Overview of the qualitative scoring per reader per sequence on a patient level (*n* = 47) with mean values of scoring, Wilcoxon signed-rank test (two-tail probabilities)

	Reader 1			Reader 2		
Assessment	BLADE T2-weighted TSE	T2-weighted TSE	*p* value	BLADE T2-weighted TSE	T2-weighted TSE	*p* value
Overall quality [scale 1–5]	4.17	4.04	0.19	4.09	4.09	1
Artefacts (ghosting) [scale 1–5]	4.40	3.83	<0.0001	4.42	3.78	<0.0001
Sharpness of uterine contours [1–5]	4.02	3.89	0.303	3.98	3.83	0.238
Visibility of fluid [0/1]	0.25	0.70	=0.0001	0.26	0.73	<0.0001
Overall contrast [scale 1–5]	3.72	4.77	<0.0001	3.66	4.79	<0.0001
Preferred sequence	19.1 %	59.6 %	=0.0065	14.9 %	53.2 %	=0.0056

### Description of artefacts

**Fig. 2:**
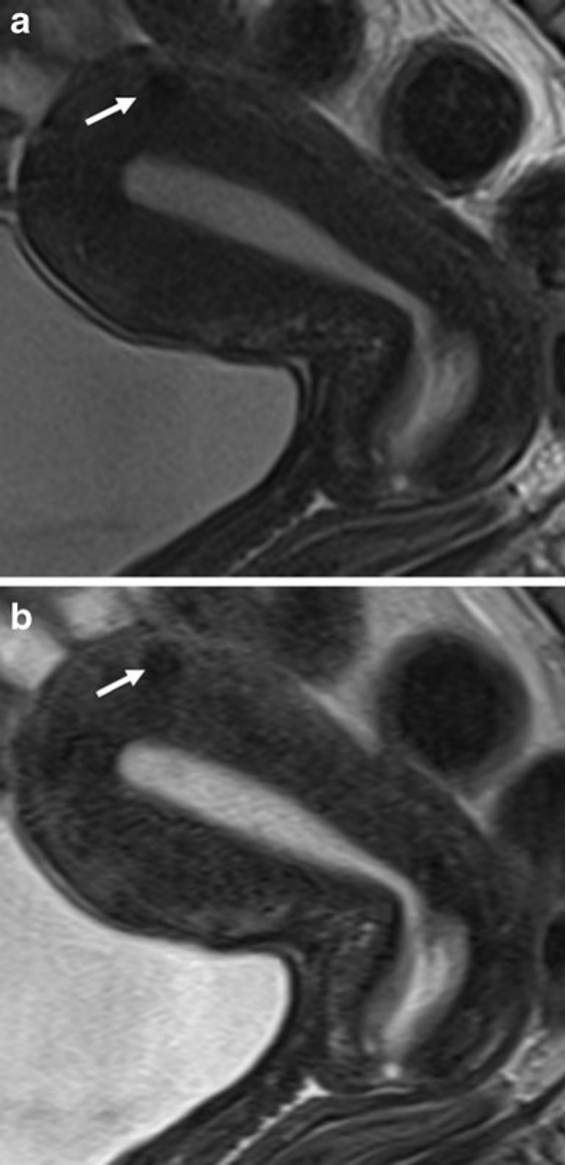
MRI of the normal anatomy of the uterus and cervix in a pre-menopausal 43-year-old patient referred to MRI for the assessment of an adnexal lesion (not shown). A small intramural leiomyoma is depicted (*arrows*). **a** Sagittal T2-weighted BLADE sequence. **b** Sagittal T2-weighted conventional TSE sequence. The uterine zonal anatomy (endometrium, junctional zone, myometrium) and the dorsal aspect of the bladder wall are more sharply delineated on the BLADE image (**a**); however, SNR and CNR are higher on the FSE image in **b**

**Fig. 3:**
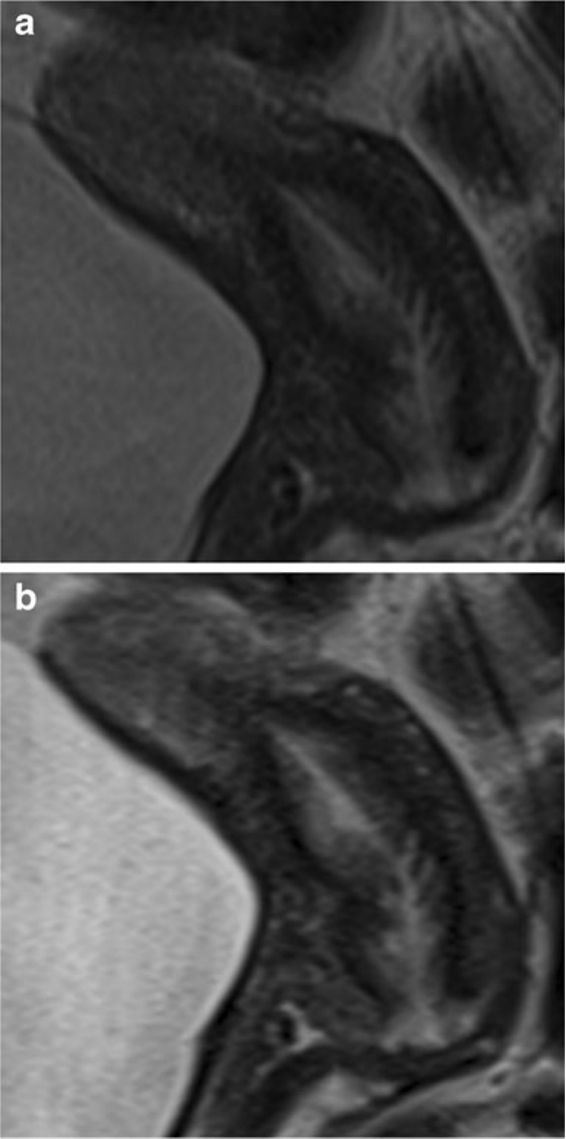
MRI of the normal anatomy of the cervix in a pre-menopausal 35-year-old patient with endometriosis. **a** Sagittal T2-weighted BLADE sequence. **b** Sagittal T2-weighted conventional TSE sequence. The cervical mucosa and stroma are more sharpely delineated on the BLADE image (**a**); however, SNR and CNR are higher on the conventional TSE image in **b**

**Fig. 4:**
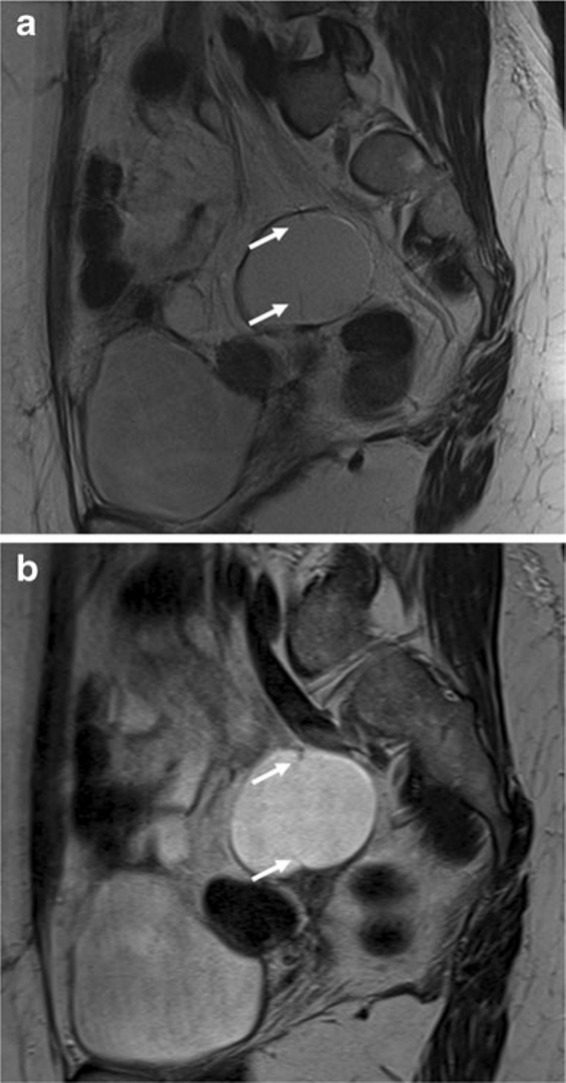
MRI of a 48-year-old patient with histologically confirmed sactosalpinx of the left side. **a** T2-weighted BLADE sequence. **b** T2-weighted conventional TSE sequence. The internal septum within fluid-containing lesion (*arrows*) is more sharply delineated on the BLADE image and there are fewer motion artefacts in the surrounding pelvic structures (**a**); however, SNR and CNR are higher on the conventional TSE image in **b**

In conventional T2-weighted TSE ghosting artefacts appeared mostly in the phase-encoding direction across the entire pelvic ROI. In BLADE images, ghosting artefacts were reduced, and when present appeared as streak artefacts. It is noteworthy that BLADE not only provided a reduction of ghosting artefacts but also an improved delineation of anatomical details in the central part of the images (Figs. 2, 3, and 4) distant to possible aliasing artefacts.

### Quantitative analysis

**Fig. 5:**
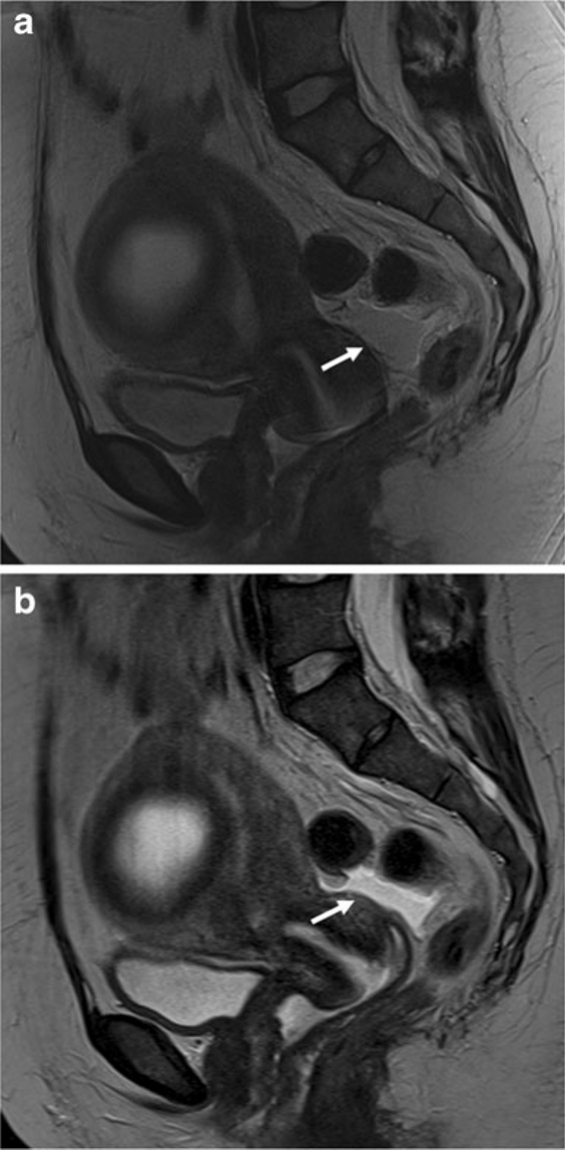
MRI in a 37-year-old patient with necrotic intramural leiomyoma. **a** T2-weighted BLADE sequence. **b** T2-weighted conventional TSE sequence. Due to the lower SNR and CNR and lower TE, the free fluid in the pouch of Douglas (*arrows*) is less obvious on the BLADE sequence. This could result in diagnostic misinterpretations

Both SNR (mean ± STD: conventional TSE 57.5 ± 37.7; BLADE 16.6 ± 12.2) and CNR (mean ± STD: conventional TSE 40.4 ± 33.5; mean ± STD: BLADE 7.2 ± 8.8) were significantly higher on the conventional T2-weighted TSE compared with BLADE (*p* < 0.0001, Figs. 2, 3, 4, and 5). The lower SNR and CNR in BLADE hampered recognition of small amounts of free fluid in the small pelvis, i.e. the pouch of Douglas (Fig. 5). Fluid in the pouch of Douglas was described in 33 (reader 1) and 34 (reader 2) cases on the conventional TSE but only in 12 cases (both readers) using BLADE TSE.

## Discussion

In this prospective comparative study we confirmed both a significant reduction of image artefacts and improved depiction of the zonal anatomy of the uterus using the T2-weighted BLADE TSE technique in comparison with conventional T2-weighted TSE. While no ghosting artefacts were present on the BLADE images, streak artefacts were visible around the bladder and abdominal wall. Although abdominal and/or femoral adipose tissue occasionally aliased into the periphery of the FOV as reported previously for the brain [4], kidneys [12] or female pelvis [6, 8], this did not significantly hamper diagnostic efficacy. In cases where gynaecological lesions are present in the centre of the FOV away from aliasing artefacts, BLADE images would allow better evaluation of these solid lesions and thus might improve diagnostic confidence (Figs. 2, 3, and 4), especially in case of cervical lesions.

The reduction of motion artefacts was observable with the BLADE technique despite the systematic use of a spasmolytic agent in our clinical set-up, demonstrating BLADE’s effectiveness. Besides being less sensitive to intestinal peristaltic motion as reported previously [3, 6, 8, 9, 13], BLADE MRI techniques allow the suppression of other motion-related ghosting artefacts. This corroborates previous results from Lane et al. [6], Fujimoto et al. [8] and also Haneder et al. [9], where centrally positioned pelvic organs (except for the bladder) were best depicted when BLADE was used in conjunction with an anti-peristaltic agent. The combination of the BLADE technique with an anti-peristaltic agent appears to yield a synergistic effect, although further clinical proof is needed in gynaecological pathologies.

To the authors’ knowledge the significantly reduced CNR for pelvic lesions on BLADE images compared with conventional T2-weighted TSE images has not yet been reported in protocols with matched principal sequence parameters (TSE approach, acquisition time, TR, spatial resolution). In our comparative study, the overall image contrast was reduced when using BLADE. This might be explained with the non-uniform T2-weighting along the width of the blade, i.e. the phase-encoding direction with contrast predominantly determined by low-frequency data as described by Pandit et al. [14]. The reduced SNR and CNR in BLADE images occurred despite more frequent sampling of the k-space centre. This was likely because the bandwidth in BLADE was twice as large as in conventional TSE (260 Hz per pixel in BLADE compared with 130 Hz per pixel in conventional TSE). Another difference between the sequences was the lower angle used in BLADE (145°) compared with conventional TSE (170°) for the radiofrequency refocusing pulses in the TSE echo train; this may have resulted in reduced signal and T2-weighting in the BLADE images. Indeed, conventional T2-weighted TSE provided a significantly better visibility of free pelvic fluid (Fig. 5) or cystic structures (i.e. a relatively higher effective T2 weighting) in numerous cases (21 and 22 for both readers, respectively). The slightly shorter echo-time of BLADE (TE = 86 ms BLADE vs TE = 101 ms conventional TSE) does not appear to explain these encountered differences of fluid visibility or contrast. Both sequences were used as proposed by the vendor. Increasing the BLADE TE to the value used in the conventional TSE sequence did not improve image quality and prolonged image acquisition time, and was therefore not evaluated further.

Both readers preferred T2-weighted conventional sequences, despite the reduction of movement and ghosting artefacts provided by BLADE. This may be related to the importance of the presence of free fluid in the pelvis as an important early sign of infection, trauma, carcinomatosis or inflammation. The readers’ preference for conventional TSE suggests that, in addition to the reduction of motion artefacts, image contrast must also considered when developing BLADE protocols for female pelvic MRI. These considerations may partly explain why differing sequence parameters were implemented in the recently published study by Fujimoto et al. [8] covering the same anatomical region. In another recent publication comparing Cartesian TSE versus BLADE algorithm image quality of the pelvic organs was rated superior with BLADE [6, 9]. Nevertheless, comparing the contrast on the figures corroborates our results with less contrast on BLADE, even though this was not quantified in this earlier retrospective study. Our results indicate that BLADE improves anatomic depiction and image quality thanks to less movement artefact, but at the expense of CNR of cystic structures or visibility of free pelvic fluid. From a diagnostic point of view, the distinctive MRI properties of cystic structures are of primary importance when performing female pelvic imaging. Typically, the watery liquid in more serous cysts is signal intensive on T2-weighted imaging, similar to urine, and hypointense on T1-weighted images, while fluid in mucinous cysts can be thicker or even haemorrhagic, with differing imaging patterns leading to higher signal on T1-weighted images and/or lower signal intensity on T2-weighted images. The BLADE-dependant reduction of CNR might therefore not only decrease visibility of non-malignant ascites, fluid collections or other non-solid structures but also hamper characterisation of the content of cystic structures. Whether this CNR reduction could lead to diagnostic problems in daily clinical setting would need to be investigated in further studies.

Indeed, the general features of liquid-containing loculations or sepatations from adenocarcinomas or other epithelial malignancies in relation to benign serous cysts or simple functional cysts such as ovarian ones might be less distinguishable. Thus, when using BLADE techniques one must be aware that the typical hallmark of mucinous tumours with different signal intensity on T2-weighted MR images might be less pronounced. Additional use of contrast showing enhancement of solid tumour components besides including conventional non-Cartesian sampled T1- and T2-weighted images into the protocol are advocated to facilitate the diagnosis and to avoid pitfalls [15]. Again, when there is concern for an indeterminate cystic lesion, the algorithmic approach with the inclusion of additional sequences allowing characterisation by their dominant signal characteristics on the standard T1-, T1-/T2-fat saturated and T2-weighted sequences must be regarded as necessary [1, 2].

In a clinical setting, the prolonged reconstruction time of BLADE is a practical drawback which must be considered. Post-processing times are dependent on the size of the raw data and are influenced by technical parameters such as blade width, coverage or parallel imaging factors, and duration of the motion correction algorithm. The post-processing interval can be used for other acquisitions, such as T1-weighted pulse sequences. However, this is impractical when the planning of subsequent sequences and their geometry rely on the BLADE images [2]. It is expected that the reconstruction time will decrease in future systems equipped with faster computers using more efficient algorithms.

Our study had several limitations. Firstly, the study was focused on qualitative and quantitative criteria describing and analysing image quality and contrast parameters. Diagnostic accuracy was not determined for the two sequences in this comparison but was also not reported in any of the previous publications. Secondly, the differing sequence parameters such as the differing bandwidth, flip-angle, voxel sizes and TE values of the two pulse sequences may be a limitation when comparing SNR and CNR values. However, the sequences were used as implemented by the vendor, and the acquisition times were quite similar. Besides that, the slightly shorter TE of BLADE (86 ms compared with 101 ms for conventional TSE) does not appear to explain the quantitative contrast differences encountered. Because an increase in the BLADE TE would have extended the acquisition time, the effect of using a longer TE was not tested systematically. Finally, we did not evaluate the performance of BLADE without the use of an anti-peristaltic agent. This decision was made for clinical reasons, to provide the best diagnostic result to our patients. The systematic suppression of peristaltic motion using glucagon shortly before acquisition of the sequences of interest may have contributed to the superior image quality obtained in this study compared with previous studies in the pelvic region [6–9].

## Conclusion

BLADE provides a reduction of motion artefacts and clearer delineation of uterine zonal anatomy relative to conventional TSE. However, these advantages come at the expense of reduced SNR and CNR hampering visibility and MR pattern recognition of fluid and cystic structures. This new technique may be useful in selected cases with uterine pathologies and in cases where severe motion artefacts may be problematic using conventional T2-weighted pulse sequences. However, due to the issues described, we would not currently recommend general replacement of conventional T2-weighted TSE sequences by BLADE in the female pelvis.
